# Reexpansion Pulmonary Edema Following Postoperative Thoracentesis After Combined Coronary Artery Bypass Grafting and Mitral Valve Surgery: A Case Report

**DOI:** 10.7759/cureus.115003

**Published:** 2026-08-22

**Authors:** Amanda Sieverts, Grace Godfrey, Tahira Awan, Jessica L Jacob

**Affiliations:** 1 Internal Medicine, Touro University Nevada, Henderson, USA; 2 Pulmonary/Critical Care, Valley Hospital Medical Center, Las Vegas, USA

**Keywords:** acute hypoxemic respiratory failure, cardiothoracic surgery, coronary artery bypass grafting, re-expansion pulmonary edema, thoracentesis

## Abstract

Reexpansion pulmonary edema (REPE) is an uncommon but potentially life-threatening complication following rapid evacuation of air or fluid from the pleural space. Diagnosis can be challenging in postoperative cardiothoracic patients because clinical and radiographic findings may overlap with more common causes of acute respiratory failure, including cardiogenic pulmonary edema and fluid overload. A 55-year-old female underwent urgent triple-vessel coronary artery bypass grafting and mitral valve repair for acute ST-segment elevation myocardial infarction. Her postoperative course was complicated by bilateral pleural effusions requiring therapeutic thoracentesis on postoperative day 13. Following removal of approximately 800 mL of serosanguineous fluid, she developed acute dyspnea, worsening hypoxemia, and rapidly escalating oxygen requirements. Repeat chest imaging demonstrated new bilateral pulmonary infiltrates without pneumothorax, raising concern for REPE. She was managed with high-flow oxygen therapy, intensive care monitoring, and supportive care, with gradual improvement in respiratory status. This case illustrates the importance of considering REPE in postoperative cardiac surgery patients who develop acute respiratory deterioration after thoracentesis, even after relatively small-volume fluid removal. Recognition of the temporal relationship between pleural drainage and respiratory decline is critical for early diagnosis and avoidance of unnecessary evaluation for alternative postoperative complications.

## Introduction

Reexpansion pulmonary edema (REPE) is a rare and potentially fatal complication following the rapid evacuation of fluid or air from the pleural space [1]. Reported incidence rates are less than 1%; however, despite its low incidence, REPE conveys significant clinical importance because of its potential for rapid respiratory decompensation and historically reported mortality rates as high as 20% in severe cases [2]. The pathogenesis is multifactorial, involving disruption of the alveolar-capillary membrane, loss of functional surfactant, and ischemia-reperfusion injury caused by inflammatory cytokines following rapid pulmonary reexpansion. Known risk factors include rapid evacuation and prolonged underlying lung collapse. However, emerging clinical evidence indicates that drainage volume alone may not reliably predict REPE risk and that no absolute “safe” volume threshold exists [1-3].

The presentation of REPE in the immediate post-cardiac surgery setting poses a distinct diagnostic and therapeutic challenge. Patients undergoing combined open coronary artery bypass grafting (CABG) and valve surgery frequently experience perioperative fluid shifts, systemic inflammatory response syndrome secondary to cardiopulmonary bypass (CPB), and postoperative pleural effusions [4,5]. In these post-cardiopulmonary surgical patients, distinguishing REPE from acute left ventricular failure, fluid overload, or severe pneumonia is difficult due to similar radiographic and clinical features.

Here, we present a case of a 55-year-old female who presented with acute hypoxemic respiratory failure consistent with REPE following right-sided thoracentesis, 13 days after undergoing urgent triple-vessel CABG and mitral valve repair on CPB. This case highlights the heightened vulnerability of post-cardiopulmonary surgery patients to abrupt intrapleural pressure changes and discusses critical management strategies in the ICU.

## Case presentation

A 55-year-old Korean female with a past medical history of essential hypertension presented to the emergency department following an acute onset of severe substernal chest pain, nausea, and dyspnea while driving home from work. Upon emergency medical services arrival, the patient was hypoxemic, with an oxygen saturation (SpO₂) of 78% on room air. An initial electrocardiogram demonstrated ST-segment elevations in lead aVL and the anterior leads, prompting activation of an ST-segment elevation myocardial infarction code. Emergent coronary angiography showed complex multivessel coronary artery disease.

The patient subsequently underwent urgent surgical intervention (designated as day 0), including triple-vessel CABG and concurrent mitral valve repair utilizing standard CPB and cardioplegia arrest. Her early postoperative course was complicated by tachy-brady syndrome, requiring the implantation of a dual-chamber permanent pacemaker on postoperative day 9. Transthoracic echocardiography following surgery demonstrated a preserved left ventricular ejection fraction of 55% without gross regional wall motion abnormalities or residual mitral regurgitation. Following clinical stabilization, her chest tube was removed on postoperative day 10, and she was successfully stepped down from the ICU to the medical floor.

**Figure 1:**
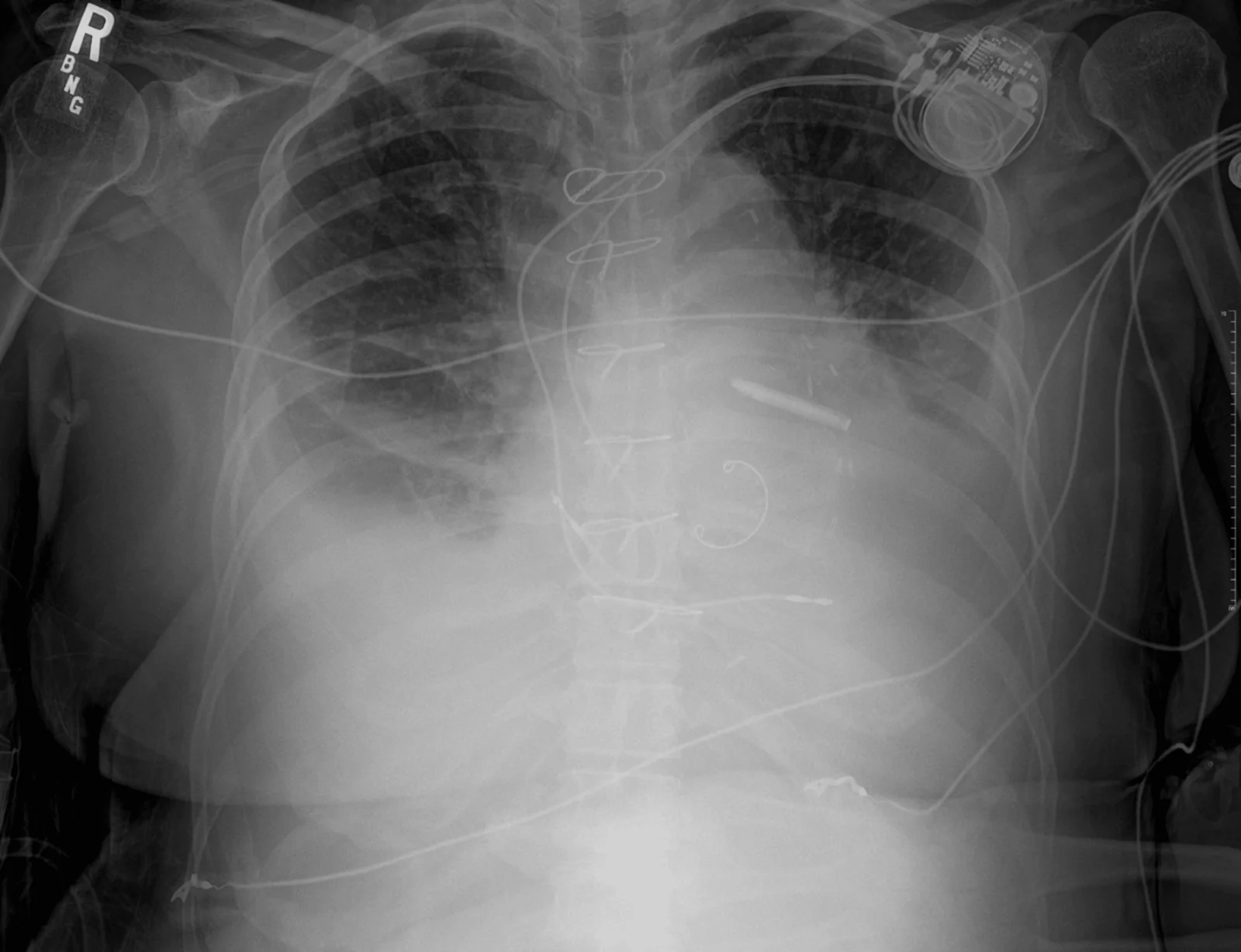
Postoperative chest radiograph on postoperative day 11 demonstrating moderate bilateral pleural effusions with baseline interstitial opacities and retrocardiac consolidation prior to thoracentesis.

**Figure 2:**
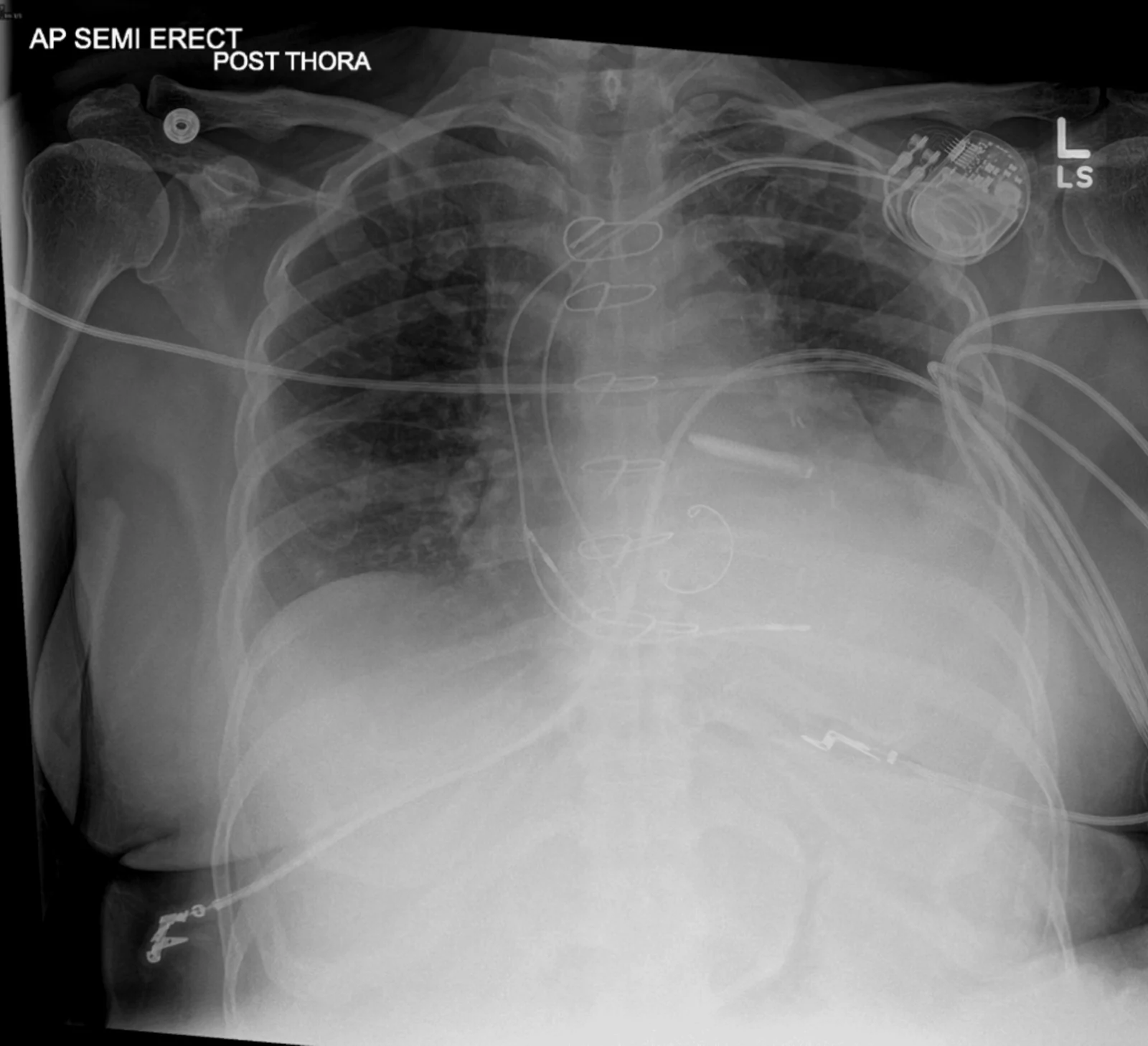
Post-thoracentesis chest radiograph on postoperative day 13 showing an interval reduction in the right-sided pleural effusion with no evidence of pneumothorax.

On postoperative day 11, a routine postoperative chest radiograph demonstrated bilateral pleural effusions with interstitial opacities and retrocardiac consolidation (Figure 1). Subsequent chest ultrasound confirmed moderate bilateral pleural effusions. Despite routine postoperative fluid management, the patient experienced persistent dyspnea and restricted ventilatory effort attributed to persistent moderate-to-large bilateral pleural effusions. On postoperative day 13, a right-sided therapeutic thoracentesis was performed at the bedside via manual gravity drainage to achieve mechanical decompression and facilitate lung reexpansion. Approximately 800 mL of serosanguineous fluid was evacuated over 20 minutes without active negative pressure suction or intrapleural pressure measurement; no pleural fluid studies were ordered. An immediate post-procedure chest radiograph demonstrated an interval reduction in the right-sided pleural effusion without evidence of pneumothorax (Figure 2). Shortly following the procedure, the patient developed acute worsening shortness of breath accompanied by persistent coughing. The patient’s oxygen requirements increased significantly following the procedure; her SpO₂ was initially 91% on 3 L nasal cannula and then 84% on 15 L non-rebreather. Because of escalating oxygen requirements, she was transferred back to the ICU for close hemodynamic and respiratory monitoring.

**Figure 3:**
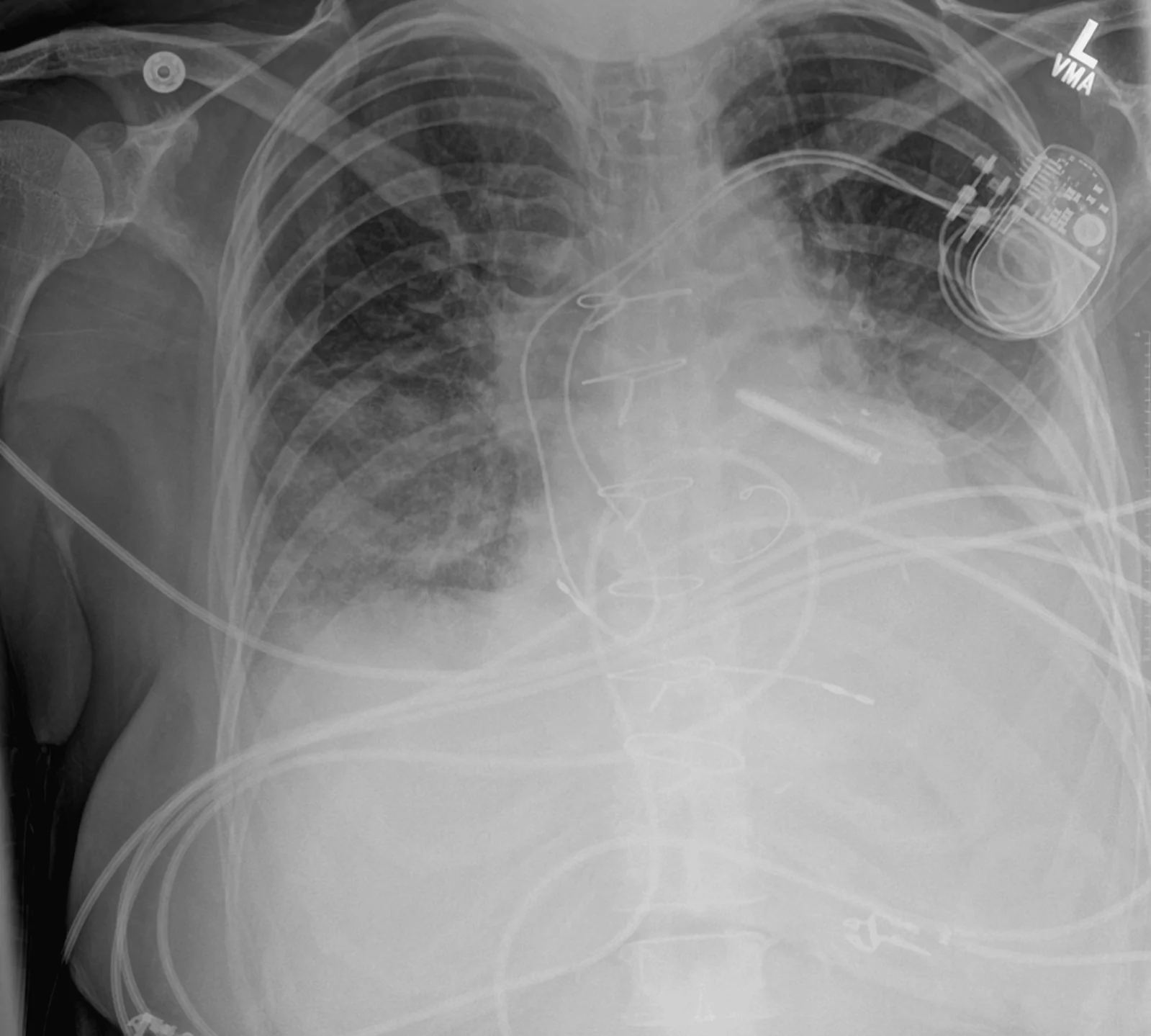
Repeat chest radiograph two hours post-thoracentesis revealing new, prominent bilateral pulmonary infiltrates consistent with suspected REPE following right-sided thoracentesis. REPE, reexpansion pulmonary edema

Repeat chest radiography obtained two hours post-procedure (Figure 3) revealed new, prominent bilateral pulmonary infiltrates superimposed on her baseline findings. Serum laboratory testing at the time of ICU transfer demonstrated stable serum creatinine (0.9 mg/dL), normal electrolytes, and a leukocytosis of 11.2 × 10³ /μL. Given the acute onset of dyspnea and cough immediately during fluid evacuation, rapid radiographic progression within two hours, and lack of systemic fluid overload on exam, a working diagnosis of suspected REPE was established.

**Figure 4:**
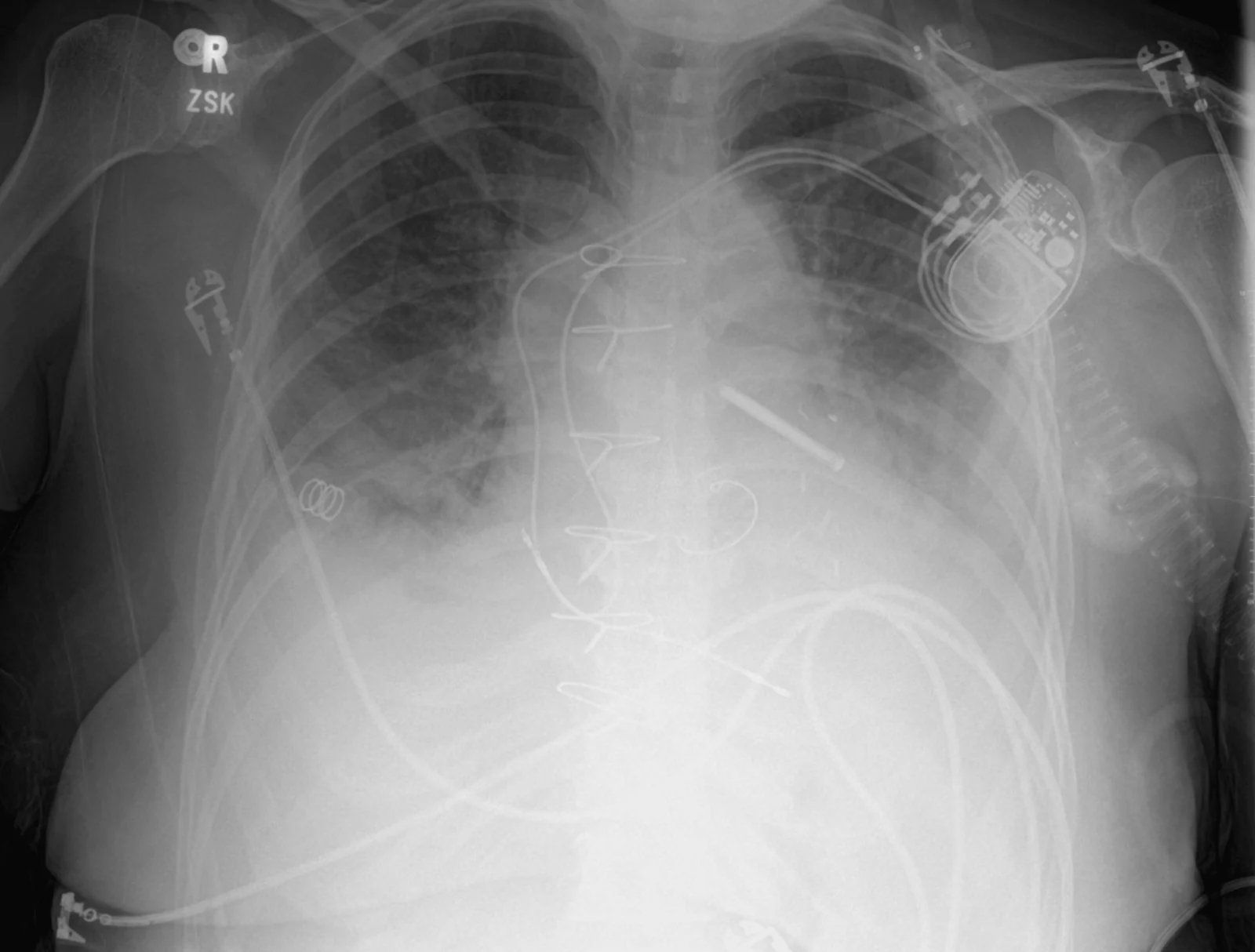
Follow-up chest radiograph 24 hours post-thoracentesis demonstrating interval improvement and stabilizing bilateral pulmonary infiltrates following ICU support and IV diuresis.

The patient was managed with supportive care, including high-flow oxygen therapy and IV furosemide (20 mg). Over the subsequent 24-48 hours, her respiratory status gradually improved, with decreasing oxygen requirements. Follow-up chest radiography 24 hours post-procedure (Figure 4) demonstrated persistent but improving bilateral pulmonary infiltrates and pleural effusions, with overall improvement in pulmonary edema compared to prior imaging.

Given the striking temporal relationship between fluid drainage and respiratory decompensation, along with rapid imaging progression and subsequent improvement with supportive management, the patient’s clinical picture was most consistent with REPE, although this cannot be definitively confirmed given baseline radiographic abnormalities. She was successfully weaned back to room air and stepped down from the ICU.

## Discussion

This case illustrates the diagnostic challenge of REPE occurring after therapeutic thoracentesis in the early postoperative period following combined CABG and mitral valve surgery. Although REPE is a recognized complication of pleural drainage, it has rarely been reported after cardiac surgery. Postoperative cardiac patients present a challenging diagnostic setting because more common etiologies of acute hypoxemic respiratory failure, including cardiogenic pulmonary edema, postoperative fluid overload, and pneumonia, frequently produce overlapping clinical and radiographic findings.

Several aspects of this patient’s clinical course favored REPE over these alternative etiologies. Most notably, she experienced abrupt respiratory deterioration immediately following thoracentesis after previously showing a stable postoperative course. Repeat chest imaging obtained less than two hours after drainage demonstrated rapidly developing bilateral pulmonary infiltrates despite successful reduction of the pleural effusion and no evidence of pneumothorax. Although cardiogenic pulmonary edema remained an important consideration given her recent myocardial infarction and cardiac surgery, preserved post-procedural ventricular function on echocardiography, normal renal function, absence of gross hypervolemia on physical exam, and immediate symptom onset during fluid evacuation strongly pointed toward a reexpansion phenomenon [4]. Nevertheless, because baseline interstitial opacities were present prior to thoracentesis, a contribution from underlying postoperative fluid retention cannot be completely excluded.

An additional clinically important feature of this case is that REPE developed after the removal of approximately 800 mL of pleural fluid, a volume significantly lower than the traditional threshold of 1-1.5 L, which has historically been associated with increased risk [6,7]. While limiting drainage volume has traditionally been recommended, recent literature demonstrates that no absolute “safe” threshold exists and that individual patient factors heavily dictate risk [6]. Our patient’s presentation supports this emerging understanding and emphasizes that clinicians should maintain a high index of suspicion for REPE, even after relatively small-volume thoracentesis.

Recent cardiac surgery utilizing CPB and cardioplegia likely increased this patient’s underlying susceptibility to reexpansion injury. CPB is known to induce a systemic inflammatory response characterized by endothelial activation, increased pulmonary vascular permeability, and pulmonary microvascular dysfunction [8]. These postoperative physiologic changes, together with pleural effusions, impaired lymphatic drainage, and altered pulmonary compliance, may have increased this patient’s susceptibility to pulmonary capillary leak following rapid lung reexpansion. Although a causal relationship cannot be established from a single case, these mechanisms provide a plausible explanation for why clinically significant REPE developed despite removal of only approximately 800 mL of pleural fluid [8].

The occurrence of REPE in the postoperative cardiac surgery population has rarely been described. Published cases have largely involved pulmonary reexpansion during CPB or minimally invasive cardiac surgery requiring one-lung ventilation rather than therapeutic thoracentesis performed during the postoperative recovery period. Prakasa and Alatas described unilateral REPE following minimally invasive atrial septal defect repair, whereas Alkhulaifi et al. reported REPE immediately after routine CABG surgery despite an otherwise uncomplicated operation [9,10]. In contrast, our patient developed REPE 13 days after combined CABG and mitral valve surgery, immediately following thoracentesis for a postoperative pleural effusion. To our knowledge, no prior reports have specifically described REPE following postoperative thoracentesis for pleural effusion after combined CABG and mitral valve surgery. This case therefore expands upon the limited existing literature by demonstrating that postoperative cardiothoracic surgery patients may remain susceptible to reexpansion injury beyond the immediate operative period and that thoracentesis itself may serve as a precipitating event.

Another noteworthy aspect of this case was the development of bilateral pulmonary infiltrates. REPE most commonly presents in the ipsilateral lung; however, bilateral involvement has been described and is generally associated with more severe disease [2,11]. Proposed mechanisms include the release of systemic inflammatory mediators and diffuse pulmonary endothelial injury, rather than injury confined solely to the reexpanded lung. In postoperative cardiothoracic surgery patients, the inflammatory response associated with CPB may further contribute to pulmonary capillary leak, making recognition of this atypical radiographic presentation particularly important [11].

Management of REPE remains primarily supportive [2]. Because the initial presentation closely resembles cardiogenic pulmonary edema, distinguishing between these entities can be particularly challenging in patients recovering from cardiac surgery. Diuretics are frequently administered when the diagnosis is uncertain because postoperative patients may simultaneously be at risk for volume overload and cardiogenic pulmonary edema; however, the benefit of diuresis in isolated REPE remains uncertain. Supplemental oxygen therapy, close hemodynamic monitoring, and appropriate escalation of respiratory support remain the cornerstones of treatment [2]. Fortunately, our patient improved with high-flow oxygen therapy, intensive care monitoring, and supportive management without requiring mechanical ventilation.

## Conclusions

This case emphasizes that REPE should remain an important diagnostic consideration in any patient who develops acute respiratory deterioration shortly after thoracentesis, regardless of the volume of pleural fluid removed. Recognition of the temporal relationship between pleural drainage and respiratory decompensation is critical, particularly in postoperative cardiothoracic patients where more common complications may initially appear more likely. This case highlights that postoperative cardiac surgery patients remain at risk for REPE beyond the immediate operative period and that thoracentesis-related respiratory deterioration should prompt consideration of this rare but potentially fatal complication.
